# Effects of Food Components That Activate TRPA1 Receptors on Mucosal Ion Transport in the Mouse Intestine

**DOI:** 10.3390/nu8100623

**Published:** 2016-10-10

**Authors:** Linda J. Fothergill, Brid Callaghan, Leni R. Rivera, TinaMarie Lieu, Daniel P. Poole, Hyun-Jung Cho, David M. Bravo, John B. Furness

**Affiliations:** 1 Department of Anatomy & Neuroscience, University of Melbourne, Parkville VIC 3010, Australia; b.callaghan@unimelb.edu.ac (B.C.); leni.rivera@deakin.edu.au (L.R.R.); Daniel.Poole@monash.edu (D.P.P.); 2 Metabolic Research Unit, School of Medicine, Deakin University, Geelong VIC 3216, Australia; 3 Monash Institute of Pharmaceutical Sciences, Monash University, Parkville VIC 3052, Australia; Tinamarie.lieu@monash.edu; 4 Biological Optical Microscopy Platform, University of Melbourne, Parkville VIC 3010, Australia; hcho@unimelb.edu.au; 5 In Vivo Animal Nutrition & Health, Talhouët, Saint-Nolff 56250, France; david.bravo@pancosma.ch; 6 Florey Institute of Neuroscience and Mental Health, Parkville VIC 3010, Australia

**Keywords:** micronutrients, enteroendocrine cells, intestine, serotonin, transepithelial transport, transient receptor potential A1

## Abstract

TRPA1 is a ligand-activated cation channel found in the intestine and other tissues. Components of food that stimulate TRPA1 receptors (phytonutrients) include allyl isothiocyanate, cinnamaldehyde and linalool, but these may also act at other receptors. Cells lining the intestinal mucosa are immunoreactive for TRPA1 and *Trpa1* mRNA occurs in mucosal extracts, suggesting that the TRPA1 receptor is the target for these agonists. However, in situ hybridisation reveals *Trpa1* expression in 5-HT containing enteroendocrine cells, not enterocytes. TRPA1 agonists evoke mucosal secretion, which may be indirect (through release of 5-HT) or direct by activation of enterocytes. We investigated effects of the phytonutrients on transmucosal ion currents in mouse duodenum and colon, and the specificity of the phytonutrients in cells transfected with *Trpa1*, and in *Trpa1*-deficient mice. The phytonutrients increased currents in the duodenum with the relative potencies: allyl isothiocyanate (AITC) > cinnamaldehyde > linalool (0.1 to 300 μM). The rank order was similar in the colon, but linalool was ineffective. Responses to AITC were reduced by the TRPA1 antagonist HC-030031 (100 μM), and were greatly diminished in *Trpa1*−/− duodenum and colon. Responses were not reduced by tetrodotoxin, 5-HT receptor antagonists, or atropine, but inhibition of prostaglandin synthesis reduced responses. Thus, functional TRPA1 channels are expressed by enterocytes of the duodenum and colon. Activation of enterocyte TRPA1 by food components has the potential to facilitate nutrient absorption.

## 1. Introduction

Herbs or herb-derived chemicals (collectively, phytonutrients) are used in very small amounts (parts per million, i.e., grams per tonne) to flavour food and, in animal feed, and are proposed to improve efficiency of nutrient digestion [1,2,3,4,5]. Several of these herbs contain compounds that activate transient receptor potential ankyrin 1 (TRPA1) channels. These include cinnamaldehyde from cinnamon, allyl isothiocyanate (AITC), which is a pungent component in mustard, radish, horseradish and wasabi, and linalool, which is found in many plant species, including mints, laurels and citrus fruits, but is not pungent [6,7,8]. TRPA1 is also activated by polyunsaturated fatty acids [9]. A site of exposure to these food components is the mucosal lining of the gastrointestinal tract. While TRPA1 expression has largely been associated with sensory nerves, and it has roles in pain sensation and inflammation, the receptor is also expressed on a multitude of non-neuronal sites, leading to ongoing investigations into its non-nociceptive functions. TRPA1 is a non-selective cation channel whose opening depolarises cells and permits calcium entry [10]. Thus, TRPA1 agonists in food would be expected to change ion currents across the mucosa if functional TRPA1 channels are expressed in the mucosal epithelium. TRPA1 mRNA has been detected by RT-PCR in colonic crypts in rat [11], in extracts of the duodenal, ileal and colonic mucosa of mouse [12,13] and in mouse and human duodenal mucosa [14]. It has been detected by immunohistochemistry in the surface epithelium of the rat colon [11] and mouse small intestine and colon [12]. TRPA1 channels have also been reported in enteroendocrine (EEC) cells of the human, rat, and mouse intestine, with most of the *Trpa1*-expressing cells being immunoreactive for 5-hydroxytryptamine (5-HT) [13,15]. AITC caused 5-HT release from isolated EEC and from a pancreas-derived enterochromaffin cell line [15].

TRPA1 agonists increase ion secretion in the pig small intestine [16], and the rat and human colon [12], in each case the effect being resistant to the blocking of nerve conduction with tetrodotoxin (TTX), which therefore appears to be a direct effect of the agonists on the mucosal epithelium. However, AITC, cinnamaldehyde, linalool and other plant-derived stimulants of TRPA1 receptors lack specificity, an example being the immune-suppressant effect of cinnamaldehyde through the inhibition of toll-like receptor 4 [17], and whether their effects on ion secretion are mediated through TRPA1 has not been investigated.

In order to gain further insight into the intestinal effects of food components that act at TRPA1 ligand-gated ion channels, we have compared the effects of cinnamaldehyde, AITC and linalool on mucosal ion transport in the small intestine and colon from normal and *Trpa1* knockout mice and on cells transfected with *Trpa1*. We also used a TRPA1 receptor blocker to investigate the pharmacology of the responses.

## 2. Methods

### 2.1. Animals

Male C57Bl/6 wild-type mice, aged eight to 10 weeks, were housed in the Biomedical Animal Facility at the University of Melbourne. *Trpa1*-deleted mice and *Trpa1*+/+ colony-matched mice on a C57Bl/6 background [18] were housed in the Monash Animal Research Platform. The animals were provided standard chow and water ad libitum. National Health and Medical Research Council of Australia (NHMRC) ethics guidelines were followed and the procedures were approved by the University of Melbourne Animal Ethics Committee (ethical approval code No. 1312777).

### 2.2. Ussing Chamber Experiments

Isoflurane was used to anaesthetise the mice; carotid arteries and spinal cord were then severed. Distal colon and duodenum segments were removed, opened along the mesenteric border and pinned, with the external muscle included, onto Ussing chamber sliders (P2311, 0.3 cm^2^ apertures, Physiological Instruments, San Diego, CA, USA) in physiological saline (Krebs’ solution: 11.1 mM glucose, 118 mM NaCl, 4.8 mM KCl, 1.0 mM NaH_2_PO_4_, 1.2 mM MgSO_4_, 25 mM NaHCO_3_, 2.5 mM CaCl_2_, pH 7.4). The sliders were inserted into two-part chambers (EasyMount Diffusion Chambers, Physiologic Instruments) and 5 mL Krebs’ solution was added to both sides, with the mucosal Krebs’ solution containing 11.1 mM mannitol instead of glucose. The glucose is required as an energy substrate, but is replaced with mannitol in the mucosal solution to restrict active transport while maintaining osmotic balance. Solutions were kept at 37 °C and gassed with carbogen (5% CO_2_, 95% O_2_) to maintain pH.

A multichannel voltage-current clamp (VCC MC6, Physiologic Instruments) was linked to each chamber through a set of four electrodes (2 voltage sensing and 2 current passing electrodes) and agar bridges (3% agarose/3 M KCl in the tip and backfilled with 3 M KCl) installed on opposite sides of the tissue. Voltage and *I*_sc_ readings were acquired using a PowerLab amplifier and recorded using LabChart^®^ 5 (both ADInstruments, Sydney, Australia).

Tissue was left to equilibrate for 30 min before clamping the voltage to 0 V. Epithelial resistance (Ω∙cm^2^) was determined from the *I*_sc_/voltage relationship by administering 2 s pulses of 2 mV every 60 s throughout most experiments and measuring the current changes.

Concentration response curves were created by consecutively adding increasing concentrations (100 nM up to 300 µM) of TRPA1 agonists (AITC, cinnamaldehyde and linalool) to the mucosal side of the Ussing chamber. The mucosal chamber was washed 4 min after the addition of the agonist, or when the response plateaued. Antagonists (HC-030031, granisetron, SB-204070, atropine, indomethacin and TTX) were added 20 min prior to the addition of a single dose of AITC (100 µM). With the exception of the TRPA1 antagonist (HC-030031), which was added to the mucosal bath, and indomethacin, which was added to both the serosal and mucosal baths, these antagonists were applied to the serosal bath. Chambers were washed after 4 min, or when the AITC response plateaued, and again 5 min later. In order to measure the time course of AITC response, a separate set of experiments was done with a single dose of AITC (100 µM) added to the mucosal bath and not washed out.

Carbachol (100 µM) was added to the serosal bath at the end of every experiment to assess the condition of the tissue. Results were excluded if the carbachol response was less than 10 μA∙cm^−2^, which is less than baseline fluctuations that were observed. Drug-induced resistance responses are presented as changes from values before drug administration.

### 2.3. Calcium Mobilisation Assay

HEK293 cells expressing rat *Trpa1* (HEK-TRPA1 cells) as previously described [19] were used. The cells were grown in Dulbecco’s modified Eagle’s medium (DMEM; Sigma-Aldrich, Sydney, Australia) containing 10% tetracycline free fetal bovine serum, 100 U∙mL^−1^ penicillin, 100 µg∙mL^−1^ streptomycin and 50 µg∙mL^−1^ hygromycin B. To induce TRPA1 channel expression, tetracycline (0.1 µg∙mL^−1^) was added to the medium 18 h before use. Non-transfected HEK293 cells, cultured without hygromycin B, were used as negative controls. Cells were grown at 37 °C with 5% CO_2_.

For calcium measurements, cells were incubated with 2.5 µM Fura2-AM (Invitrogen, Sydney, Australia) and pluronic acid (Invitrogen; 0.01%) in HEPES buffer (138 mM NaCl, 5 mM KCl, 1.2 mM MgCl_2_, 2 mM CaCl_2_, 10 mM glucose, 10 mM HEPES, pH 7.4) for 1 h at 37 °C. A FlexStation three-plate reader (Molecular Devices, Sunnyvale, CA, USA), was used to dispense TRPA1 agonists and monitor changes in intracellular Ca^2+^. Fluorescence was measured (4 s intervals) at 340 nm and 380 nm excitation and 510 nm emission wavelengths for 120 s.

Agonists were added at 15 s, and antagonists were pre-incubated 20 min before the addition of the agonist. Data were recorded using SoftMax Pro^®^ 5.4. The mean of the peak fluorescence ratio after agonist injection minus the basal ratio was used for plotting concentration response curves as previously described [20].

### 2.4. Compounds

AITC, cinnamaldehyde, linalool, carbachol, indomethacin, TTX, atropine and SB-204070 were purchased from Sigma-Aldrich (Sydney, Australia). Granisetron was from SmithKline Beecham, Harlow, UK and HC-030031 was from Sapphire Biosciences, Melbourne, Australia. TRPA1 agonists (AITC, cinnamaldehyde and linalool) and TRPA1 antagonist (HC-030031) stock solutions were dissolved in dimethyl sulfoxide (DMSO; maximum final volume 0.3%). Stock solutions of the remaining compounds were made with distilled water. Further dilutions were made with HEPES buffer for calcium mobilisation experiments and distilled water and Krebs’ solution for Ussing chamber experiments.

### 2.5. Data Analysis

Data from both the Ussing and calcium mobilisation concentration-response experiments are presented as linear regression curves. A one-way ANOVA was used when comparing three or more experimental groups, using a Dunnetts’ post hoc test to compare groups to the vehicle control. An unpaired *t*-test was performed when comparing two groups.

pA_2_ values were calculated from concentrations of antagonists that did not depress the maximum agonist response using the Gaddum/Schild EC_50_ shift analysis using GraphPad Prism 5.0 (Graph-Pad Software, San Diego, CA, USA). Data are presented as mean ± SEM. Significance was set at *p* < 0.05.

## 3. Results

### 3.1. Effects of AITC, Cinnamaldehyde and Linalool on Ca^2+^ Mobilisation in HEK-TRPA1 Cells

**Figure 1:**
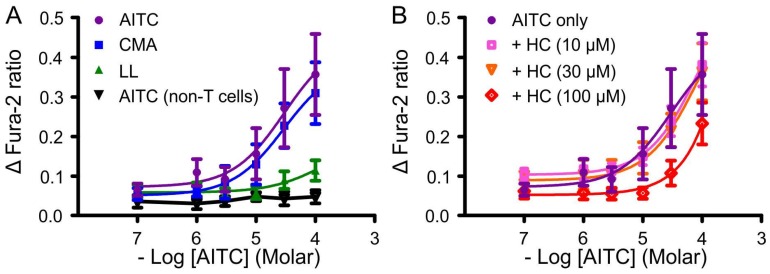
Concentration-response relationships for TRPA1 agonists and the effect of a TRPA1 receptor antagonist on HEK393 cells transfected with the *Trpa1* gene. (**A**) Calcium mobilisation (expressed as Δ Fura-2 ratio) in response to allyl isothiocyanate (AITC), cinnamaldehyde (CMA) and linalool (LL); (**B**) The inhibitory effect of graded concentrations of HC-030031 (HC) on the response to AITC in the transfected cells. Neither the agonists nor the antagonist had any effect in the HEK293 cells that were not transfected with *Trpa1*, indicated by AITC (non-T cells) in (**A**). The weak effect of linalool is reflected in the studies of mucosal function.

AITC, cinnamaldehyde and linalool increased cytoplasmic Ca^2+^ in HEK-TRPA1 cells but not in non-transfected HEK293 cells (Figure 1A, data not shown for cinnamaldehyde and linalool on non-transfected HEK293 cells). The most potent agonist at 100 µM was AITC (0.36 ± 0.10 increase in ΔFura-2 ratio, *n* = 5). The calcium response to cinnamaldehyde (100 μM) was 87% ± 22% of the AITC response (*n* = 4), and to linalool (100 μM) was 32% ± 7% (*n* = 5) of the AITC response. The TRPA1 antagonist HC-030031 (100 µM), added to the cells at least 20 min before AITC, produced a rightward shift in the concentration response curves in HEK-TRPA1 cells (Figure 1B; response at 100 µM AITC plus HC-030031 was 65% ± 15% of AITC alone, *n* = 5). At 10 and 30 μM, HC-030031 had a minimal effect on the AITC response curve (Figure 1B; response at 100 µM AITC was 107% ± 15%, *n* = 4 and 102% ± 20%, *n* = 4 of the response to AITC alone, respectively). The calculated pA_2_ for HC-030031 antagonism of AITC activation of rat TRPA1 was 4.27 ± 0.21.

### 3.2. Effects of AITC, Cinnamaldehyde and Linalool on Short Circuit Current (I_sc_)

**Figure 2:**
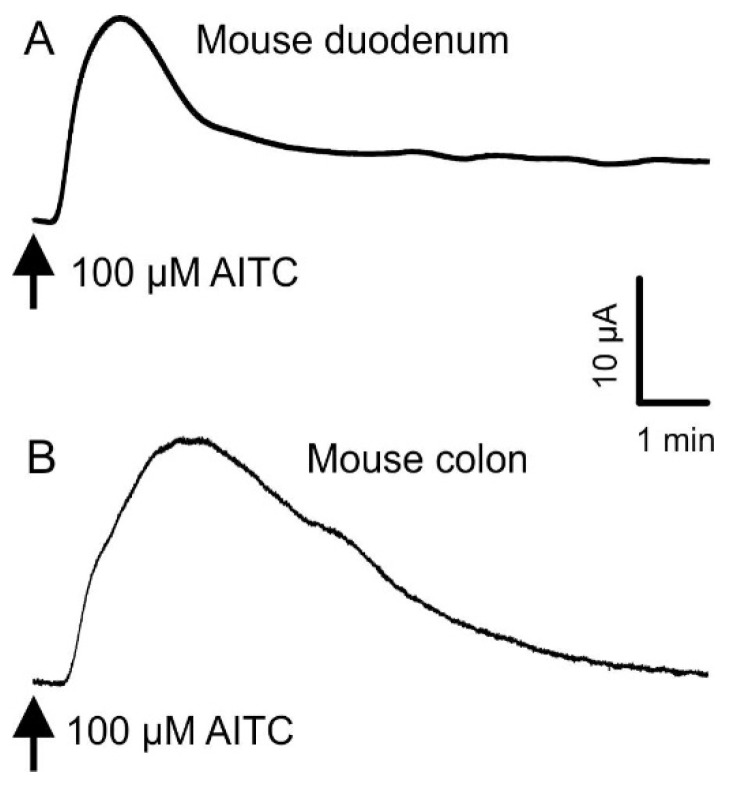
Representative traces of the short circuit current (*I*_sc_) following addition of AITC (100 µM). AITC was added to the mucosal side of the Ussing chamber mounted with wild-type mouse duodenum (**A**) and distal colon (**B**).

In these experiments, the basal resistance in the duodenum was 61.6 ± 0.8 Ω∙cm^2^ and the basal *I*_sc_ was 3.65 ± 0.38 µA∙cm^−2^ (*n* = 122). In the colon, the basal resistance was 114.0 ± 2.3 Ω∙cm^2^ and the basal *I*_sc_ was 6.02 ± 0.59 µA∙cm^−2^ (*n* = 106). TRPA1 agonists, applied to the mucosal side of mouse duodenum and colon tissue mounted in Ussing chambers, caused transient increases in the short circuit current (Figure 2). Responses to AITC (100 µM) were greater in magnitude in the colon (58.7 ± 5.1 µA∙cm^−2^, *n* = 11) than in the duodenum (21.4 ± 3.3 µA∙cm^−2^, *n* = 13). Responses to 100 µM AITC peaked between 1 and 1.4 min in the duodenum (*n* = 4) and 2 and 6.7 min in the colon (*n* = 7; Figure 2). These responses declined to 50% at 2.3 to 2.6 min and at 4.6 to 9.0 min after adding AITC to the duodenum and colon, respectively. Small, brief, transient decreases in *I*_sc_ were sometimes observed, prior to the increased *I*_sc_, following the addition of AITC, cinnamaldehyde, and linalool, 100 to 300 µM, to the duodenum but not the colon.

**Figure 3:**
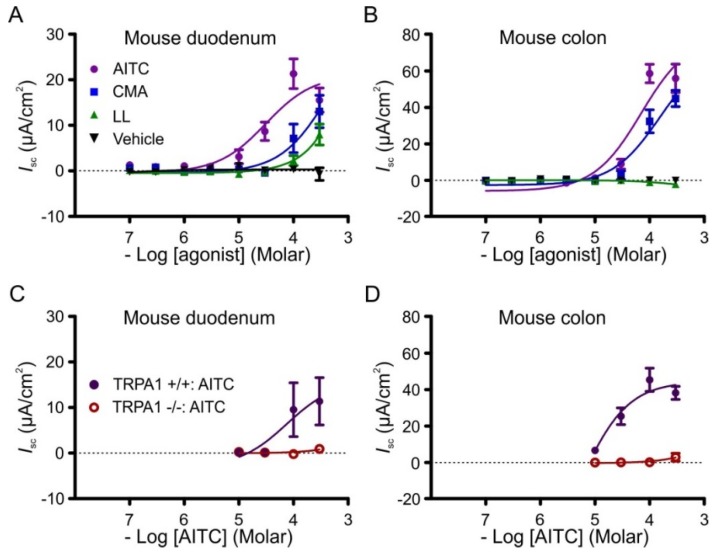
Concentration-response relationships for TRPA1 agonists on *Trpa1*+/+ and −/− tissues in Ussing chambers. Change in short circuit current (*I*_sc_) in response to allyl isothiocyanate (AITC), cinnamaldehyde (CMA) and linalool (LL) in wild-type mouse duodenum (**A**) and distal colon (**B**); Change in *I*_sc_ in response to AITC in duodenum (**C**) and distal colon (**D**) from *Trpa1* −/− mice and *Trpa1* +/+ mice from the same colony.

In cumulative concentration response determinations, the most potent agonist at 100 μM in the duodenum was AITC (21.4 ± 3.3 µA∙cm^−2^, *n* = 13), then cinnamaldehyde (7.2 ± 3.1 µA∙cm^−2^, *n* = 7) and then linalool (2.3 ± 1.1 µA∙cm^−2^, *n* = 6) (Figure 3A). In the colon only AITC (58.7 ± 5.1 µA∙cm^−2^, *n* = 11) and cinnamaldehyde (32.5 ± 0.4 µA∙cm^−2^, *n* = 8) were potent, with linalool having no effect even at 300 µM (−1.0 ± 0.4 µA∙cm^−2^, *n* = 6; Figure 3B).

### 3.3. Effects of Pharmacological Antagonism and Ablation of Trpa1 on Responses to AITC

**Figure 4:**
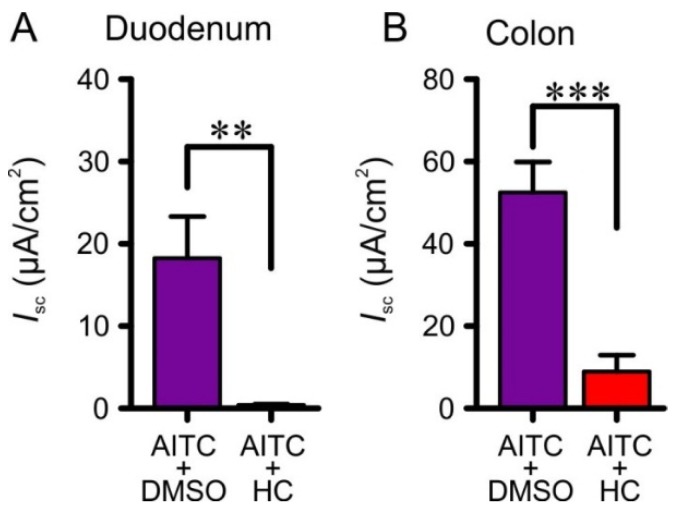
The TRPA1 receptor antagonist, HC-030031 (HC; 100 µM), reduced the effects of AITC (100 µM) in both the duodenum (**A**) and colon (**B**). *** *p* < 0.01; ** *p* < 0.05. DMSO is the vehicle for dissolving HC-030031.

In the presence of the TRPA1 antagonist, HC-030031 (100 µM), *I*_sc_ responses to AITC (100 µM) were significantly reduced in both the duodenum (18.3 ± 5.1 µA∙cm^−2^, *n* = 6, to 0.41 ± 0.12 µA∙cm^−2^, *n* = 6; *p* < 0.01) and the colon (52.6 ± 7.4 µA∙cm^−2^, *n* = 7, to 8.9 ± 4.0 µA∙cm^−2^, *n* = 5; *p* < 0.001) (Figure 4).

AITC (300 µM) was significantly less potent in *Trpa1*−/− tissues compared to controls in the duodenum (*Trpa1*+/+: 11.4 ± 5.2 µA∙cm^−2^, *n* = 4; *Trpa1*−/−: 0.9 ± 0. 8 µA∙cm^−2^, *n* = 6; *p* < 0.05) and in the colon (*Trpa1*+/+: 38.3 ± 3.6 µA∙cm^−2^, *n* = 3; *Trpa1*−/−: 3.0 ± 2.1 µA∙cm^−2^, *n* = 4; *p* < 0.001) (Figure 3C,D). In these experiments, effects in tissues from the wild-type mice (*Trpa1*+/+ mice) from the same breeding colony as the *Trpa1*−/− mice were used for comparison. Responses in the duodenum from these colony-matched *Trpa1*+/+ mice were smaller than in the C57Bl6 mice used in the remainder of this study.

**Figure 5:**
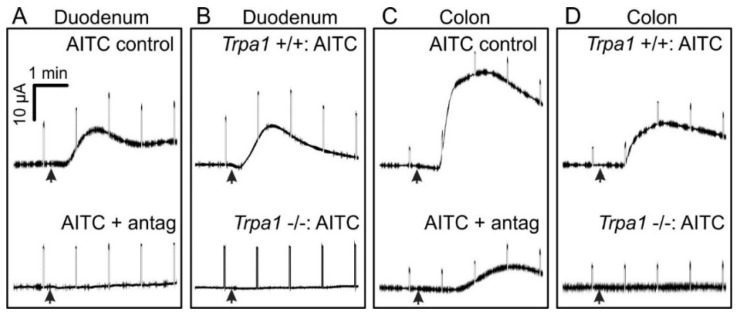
Representative traces of the *I*_sc_ following addition of AITC (100 µM) to duodenum (**A**,**B**) and colon (**C**,**D**) segments. (**A**,**C**) AITC was added after 20 min incubation with either DMSO (control) or HC-030031 (100 µM; antagonist); (**B**,**D**) AITC was added to *Trpa1*+/+ control tissue and *Trpa1*−/− tissues. Arrowheads: AITC (100 µM) added to mucosal solution. The transients that appear in these records were the responses to current pulses used to measure trans-epithelial resistance.

Representative traces of the response to AITC following incubation with HC-030031, and also in the *Trpa1* −/− and *Trpa1* +/+ tissue, are shown in Figure 5.

### 3.4. Effects of AITC, Cinnamaldehyde and Linalool on Transmucosal Resistance

**Figure 6:**
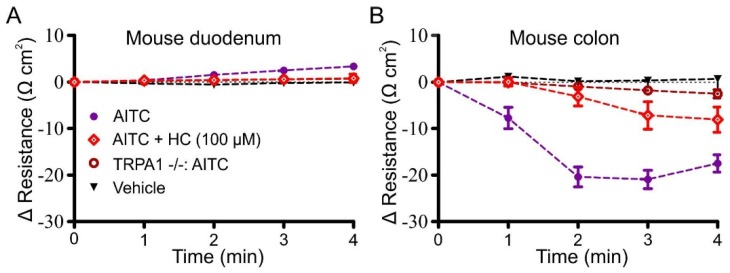
Transmucosal resistance in the first 4 min following the addition of AITC (100 µM). Comparison of the effect of AITC only, AITC following pre-incubation with HC-030031 (100 µM; HC), and AITC in *Trpa1*−/− tissues of mouse duodenum (**A**) and distal colon (**B**).

AITC (100 µM) caused a significant decrease in resistance in the colon compared to the vehicle (−21.8 ± 1.9 Ω∙cm^2^, equivalent to decreasing the initial resistance by 19.0% ± 1.7%, *n* = 11; *p* < 0.001; Figure 6B), whereas it caused a small significant increase in resistance in the duodenum (3.2 ± 0.7 Ω∙cm^2^, equivalent to 5.2% ± 1.2% of the initial resistance, *n* = 13; *p* < 0.05; Figure 6A). No other groups were significantly different to the vehicle in the duodenum. The order of efficacy of TRPA1 agonists in reducing transmucosal resistance in the colon was the same as the order of efficacy for the change in *I*_sc_. Cinnamaldehyde (100 µM) caused a significant reduction in resistance (−14.0 ± 2.3 Ω∙cm^2^, *n* = 8; *p* < 0.01), and linalool (100 µM) had little effect (4.4 ± 1.1 Ω∙cm^2^, *n* = 6; Figure 6B). The reduction in resistance in response to 100 μM AITC (−19.2 ± 3.4 Ω∙cm^2^, *n* = 7) in the colon was significantly lower when the tissue was pre-incubated with 100 μM HC-030031 (−8.0 ± 2.7 Ω∙cm^2^, *n* = 5; *p* < 0.0001), and the effect of AITC on resistance in the colon of *Trpa1*+/+ mice (−18.8 ± 0.6 Ω∙cm^2^, *n* = 3) was absent in *Trpa1*−/− tissues (−2.5 ± 0.9 Ω∙cm^2^, *n* = 4; *p* < 0.05).

### 3.5. Effects of 5-HT Receptor Blockers, Tetrodotoxin, Atropine and Indomethacin

**Figure 7:**
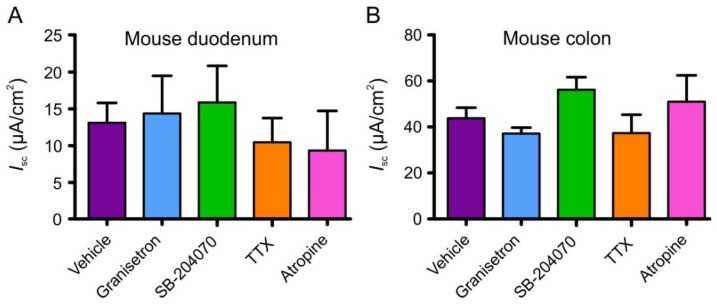
Effect of AITC (100 µM) on *I*_sc_ following a 20 min incubation period with antagonist in mouse duodenum (**A**) and colon (**B**). Tissues were incubated with granisetron, SB-204070, TTX, atropine and their vehicle (5 µL dH_2_O) for 20 min. No change in *I*_sc_ was observed in response to the AITC vehicle (5 µL DMSO) after a 20 min incubation with 5 µL added water (data not shown). No statistically significant difference was found between experimental groups using a one-way ANOVA.

Responses to AITC (100 µM) in the presence of the 5-HT_3_ receptor antagonist granisetron (1 µM; *n* ≥ 6), the 5-HT_4_ receptor antagonist SB-204070 (1 µM; *n* ≥ 8), the muscarinic acetylcholine receptor antagonist atropine (10 µM; *n* = 6), or the sodium channel blocker TTX (1 µM; *n* ≥ 7) were not significantly different to AITC alone (100 µM; *n* = 16) in either the duodenum or the colon (Figure 7). None of these compounds changed the baseline *I*_sc_.

**Figure 8:**
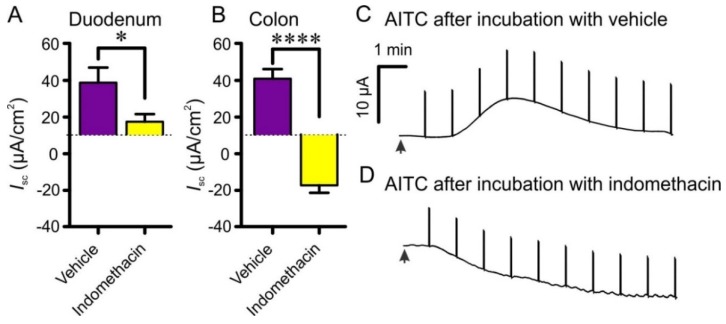
Effect of AITC (100 µM) following a 20 min incubation with indomethacin (10 µM) or its vehicle (5 µL DMSO) on the mucosal and serosal side of duodenum (**A**) and colon (**B**) segments; Representative traces of the *I*_sc_ following addition of AITC (100 µM) to colon segments following a 20 min incubation with vehicle (**C**) and indomethacin (**D**). Arrowheads: AITC (100 µM) added to mucosal solution. The transients that appear in these records are the responses to current pulses used to measure trans-epithelial resistance.

The prostaglandin synthase inhibitor indomethacin (10 µM) significantly reduced responses in the duodenum (28.7 ± 8.2 µA∙cm^−2^, *n* = 7, to 7.6 ± 4.2 µA∙cm^−2^, *n* = 7; *p* < 0.05) and reversed the effect of AITC from an increase to a decrease in the colon (30.8 ± 5.3 µA∙cm^−2^, *n* = 7, to −27.4 ± 4.1 µA∙cm^−2^, *n* = 4; *p* < 0.0001) (Figure 8). Representative traces are shown in Figure 8C,D.

## 4. Discussion

Three plant-derived TRPA1 agonists, allyl isothiocyanate (AITC), cinnamaldehyde, and linalool, which are commonly used as food additives, all caused increases in the short circuit current in the duodenum and colon of the mouse when applied to the luminal surface. The most potent of these, AITC, was used to investigate the specificity of the effect. Its action was antagonised by the TRPA1 antagonist HC-030031, and was almost completely absent in the duodenum and colon of *Trpa1*−/− mice. This indicates that the agonists act through TRPA1 receptors to stimulate transepithelial ion transport, which is linked to fluid movement across the epithelium [21,22]. The residual response in the colon of *Trpa1*−/− mice indicates that a small component of AITC action is not through the TRPA1 receptor. Stimulation of ion transport can be through a direct effect on the epithelium, or indirectly, for example through the release of hormones from EEC. Hormones, such as 5-HT, could in turn activate secretomotor neurons. We have previously found that *Trpa1* gene transcripts are expressed by 5-HT containing EEC in the duodenum, but not in the colon [13]. Other studies have detected TRPA1 protein by immunohistochemistry in enterocytes of the mucosal epithelium of the small intestine and colon of the mouse [12] and the colon of the rat [11]. 5-HT released from the EEC acts indirectly, through 5-HT_3_ and 5-HT_4_ receptors that stimulate mucosal nerve endings and activate secretomotor neurons [23,24]. Action potential conduction in these neurons is blocked by TTX [23]. Thus, if stimulation of mucosal secretion is indirect through 5-HT release from EEC and neural activation, it would be expected to be blocked by TTX and to be reduced by the 5-HT_3_ and 5-HT_4_ receptor antagonists, granisetron and SB-204070. However, none of these compounds caused any reduction of responses in the duodenum or colon. We therefore conclude that the increases in *I*_sc_ are caused by actions on enterocytes and that these actions are not indirect through activation of enteric neurons or release and action of 5-HT. As mentioned, enterocytes show immunoreactivity for TRPA1, but we did not find gene expression in these cells using in situ hybridisation [13]. It is feasible that the level of expression of the gene product and turnover of the receptor is low, and thus the amount of mRNA in individual enterocytes is below the level of detection by in situ hybridisation. However, RT-PCR reveals *Trpa1* gene expression in the duodenal and colonic mucosa of the mouse [12,13]. Thus, the present pharmacological and gene knockout data is consistent with immunohistochemical and RT-PCR observations, and allows us to conclude that there are functional TRPA1 channels on enterocytes in the mouse.

The effects of the TRPA1 agonists were reduced by the prostaglandin synthase inhibitor indomethacin, which confirms the results of Kaji et al. [11], who found that AITC-evoked secretion was reduced by piroxicam and a prostaglandin EP4 receptor antagonist. These results suggest that TRPA1 agonists stimulate prostaglandin production in the mucosa. Effects of histamine and reflexly-evoked secretion are also reduced by indomethacin [25,26]. Thus, prostaglandins might be intermediates in responses of the mucosa to a range of stimuli. In some cases, but only in the duodenum, the direction of the *I*_sc_ change was reversed after indomethacin. It is likely that this decrease in *I*_sc_ is a response to TRPA1 activation, because it occurred with all three agonists, but these occasional responses have not been investigated further.

TRPA1 can closely associate with TRPV1, for example in sensory neurons [27], and TRPV1 ligands can change the properties of the associated TRPA1 channel, tending to reduce its opening. Thus, TRPV1 agonists that occur in foods, for example capsaicin, may reduce TRPA1-mediated increases in secretion. However, these potential interactions have yet to be investigated in the intestine.

AITC and cinnamaldehyde decreased trans-mucosal resistance in colon tissue. This effect was blocked by HC-030031 and was absent in duodenal segments and in colon tissue from *Trpa1* knockout mice. Kaji and colleagues observed a similar increase in transepithelial conductance in the rat colon but not the human colon, following AITC and cinnamaldehyde, which was also inhibited by HC-030031 [10]. The underlying reason for the changes in conductance in some tissues and not others in response to TRPA1 activation is currently undetermined.

This study has verified that activation of TRPA1 channels in a recombinant system (HEK293 cells) causes an increase in the intracellular calcium concentration. Nozawa and colleagues have also shown that activation of endogenous TRPA1 channels mediates an influx of extracellular calcium in the rat pancreatic endocrine cell line, RIN14B [15]. Influx of calcium in response to Sodium Glucose Transporter 1 activation has previously been shown to contribute to the cytoskeletal rearrangements that lead to the insertion of GLUT2 (facilitative glucose transporter) in the apical membrane of enterocytes [28,29,30]. Further studies are required to ascertain if the calcium influx induced by TRPA1 activation also initiates similar mechanisms, i.e., increasing absorption of glucose in enterocytes. Micronutrients that activate TRPA1 have been reported to enhance nutrient handling efficiency [1,2,3,4,5] and this may be contributed to by a TRPA1-initiated series of events leading to greater carbohydrate absorption.

## 5. Conclusions

In conclusion, this study demonstrates functional TRPA1 expression in the mucosa of the small intestine and colon in mice, and provides a possible explanation of the mechanism through which phytonutrients acting at TRPA1 affect mucosal function. The data point to the rational use of these phytonutrients to enhance digestive efficiency.
